# Near-complete genome sequences of human enterovirus A71 genotype A (BrCr-like) isolates from hand, foot, and mouth disease patients in India

**DOI:** 10.1128/mra.01320-25

**Published:** 2026-03-10

**Authors:** Kaveri Jagtap, Pradeep Mahadev Sawant, Basavaraj Mathapati, Swagnik Roy, Vikas Sharma, Pronami Gogoi, Sivasankar Panickan, Mallika Lavania

**Affiliations:** 1 ICMR - National Institute of Virology 29620, Pune, Maharashtra, India; 2 ICMR - National Institute of Virology, Pune, East Zone, Dibrugarh, Assam, India; 3 Zoram Medical College and Hospital, Falkwan, Mizoram, India; Katholieke Universiteit Leuven, Leuven, Belgium

**Keywords:** enterovirus, whole genome

## Abstract

We report near-complete (~7.4 kb) genomes of two human enterovirus A71 (EV-A71) isolates from Rajasthan (2022) and Mizoram (2024), India. Genotyping and phylogenetic analyses placed both within genotype A (BrCr-like), expanding EV-A71 diversity associated with hand, foot, and mouth disease in India.

## ANNOUNCEMENT

Enterovirus A71 (EV-A71), a serotype of the species *Enterovirus alphacoxsackie* (family *Picornaviridae*), is a major cause of hand, foot, and mouth disease (HFMD) and can lead to severe neurological complications in children (1). Although HFMD outbreaks are reported in India (2–4), near-complete EV-A71 genome sequences are unavailable.

**Fig 1:**
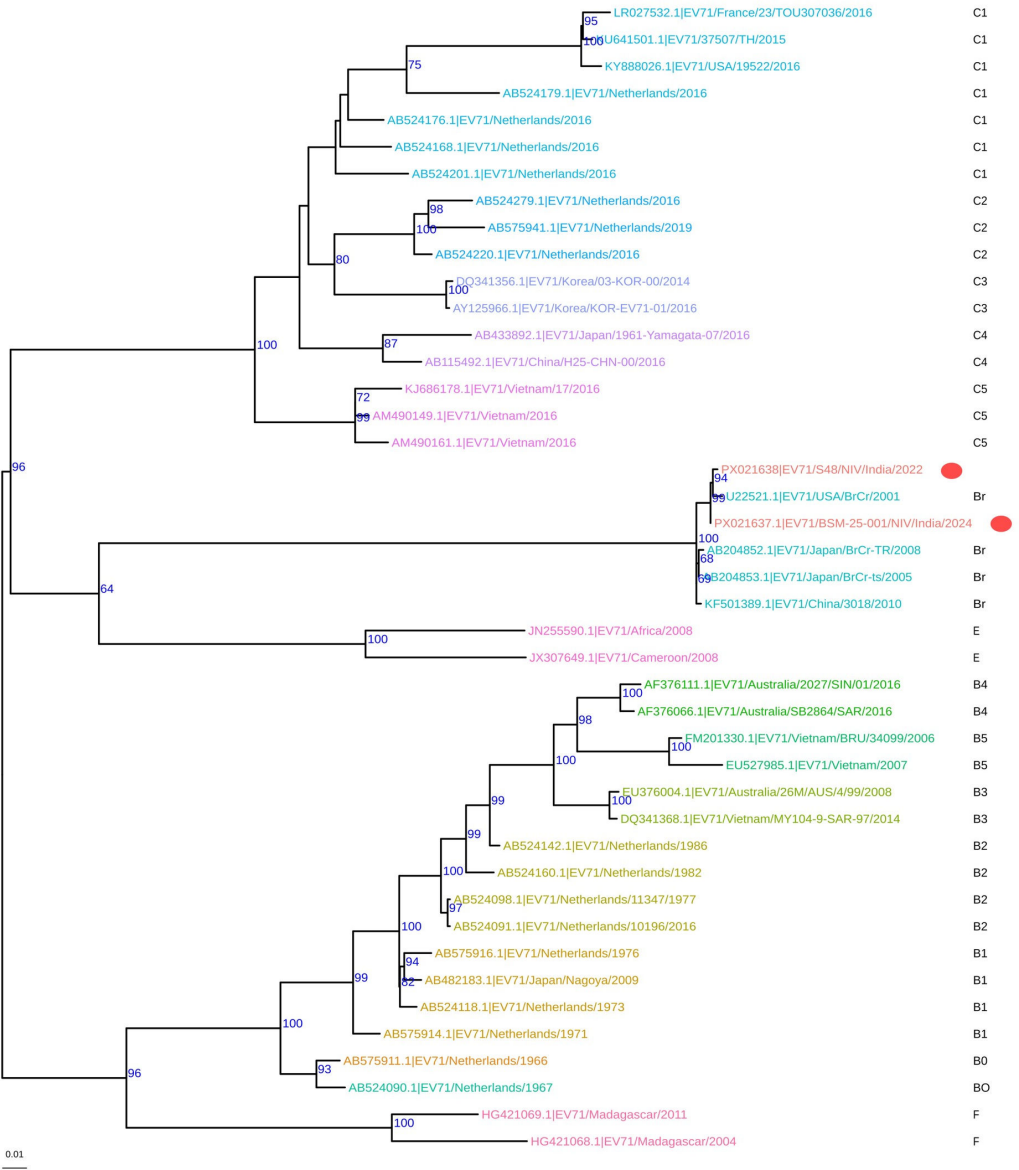
Phylogenetic tree of the EV-A71 complete VP1 gene: Sequences were aligned using MAFFT v7 (--auto --reorder --adjustdirection), manually curated, and processed using SAMtools (v1.21). The maximum likelihood phylogenetic tree was constructed using IQ-TREE (v2.0.7) (12) with the best-fit substitution model selected automatically and 1,000 bootstrap replicates. Bootstrap support values ≥60% are indicated at the corresponding nodes. The tree was visualized in ggtree (v3.10.1) (13). Sequences are color-coded according to EV-A71 genotypes, with genotype A (BrCr-like) highlighted. The two EV-A71 isolates from India are highlighted with red circles. The scale bar represents 0.01 substitutions per nucleotide site.

In this study, a vesicular swab sample (NIV2420350) was collected on 17 August 2022, from an 11-year-old male clinically diagnosed with HFMD at Sardar Patel Medical College, Bikaner, Rajasthan, during an HFMD outbreak. A second sample (NIV224291), a nasopharyngeal swab, was collected from an 8-month-old female with fever, mouth ulcers, and vesicular lesions on hands and abdomen with neurological manifestations on 5 July 2024, from the pediatrics OPD of Zoram Medical College, Mizoram, as part of virological surveillance under ICMR ad hoc project 2021_9126. The Institutional Human Ethics Committee of Zoram Medical College approved the study (Letter no. NIV/IEC/March/2023/D1). Initial screening used pan-enterovirus primers targeting the conserved 5′ UTR region (5), followed by EV-A71-specific VP1 gene amplification (6). Virus isolation was performed in rhabdomyosarcoma (RD) cells (7, 8). Briefly, 100 µL of each clinical sample was inoculated onto RD cells seeded in 24-well plates and incubated at 37 °C with 5% CO_2_ for 5–6 days. Cultures showing cytopathic effects were harvested and plaque-purified twice in Vero cells. Viral RNA was extracted from culture supernatants using the QIAamp Viral RNA Mini Kit. Sequencing libraries were prepared using the Illumina RNA Library Prep Kit (10–100 ng input; Cat. no. 20040537) and enriched using the Illumina Viral Surveillance Panel v2 (Cat. no. 20107489). Paired-end sequencing was performed on the Illumina MiSeq platform. Quality-trimmed reads (35–301 nt) were assembled *de novo* using rnaSPAdes (v4.0.0) with default parameters (9) and also mapped to the EV-A71 reference genome (AB204852.1) using BWA-MEM (version 0.7.18) with default parameters. Variants were called using BCFtools (version 1.21), and consensus sequences were generated. Genome coverage was evaluated with SAMtools (v1.21), and regions lacking coverage were masked in consensus sequences. Near-complete genome sequences of 7,407 bp (BSM-25-001; NIV2420350) and 7,406 bp (S48; NIV224291) were obtained, with average read depths of 6,856× and 14,373×, respectively, and a GC content of 47.5%. Genome annotation was performed using VAPiD (v1.6.7) with default settings (10) in conjunction with the NCBI RefSeq Viral database. VAPiD automatically selected the reference genome based on the highest BLAST similarity to the query sequence (RefSeq Viral release 219; accessed March 2024). Enterovirus Genotyping Tool with default parameters (https://mpf.rivm.nl/mpf/typingtool/enterovirus/) identified both isolates as EV-A71 genotype A (BrCr-like). A BLASTn similarity search showed 99.74–99.80% nucleotide identity and 100% query coverage with EV-A71 strain AB204852.1 (BrCr prototype strain), which belongs to genotype A and was originally isolated from Japan (11). A maximum likelihood phylogenetic analysis of the complete VP1 region using IQ-TREE v2 (ModelFinder, 1,000 ultrafast bootstrap replicates) showed strong clustering (bootstrap >99%) with strains from the United States (U22521.1), Japan (AB204852.1 and AB204853.1), and China (KF501389.1) (Fig. 1).

## Data Availability

The near-complete genome sequences of two EV-A71 isolates have been deposited in GenBank via BankIt under accession numbers PX021637 and PX021638. The corresponding raw sequence reads are available in the Sequence Read Archive (SRA) under accession numbers SRR34876838 and SRR34877665.
